# Male Mating Rate Is Constrained by Seminal Fluid Availability in Bedbugs, *Cimex lectularius*

**DOI:** 10.1371/journal.pone.0022082

**Published:** 2011-07-11

**Authors:** Klaus Reinhardt, Richard Naylor, Michael T. Siva-Jothy

**Affiliations:** 1 Department of Animal and Plant Sciences, The University of Sheffield, Sheffield, United Kingdom; 2 Animal Evolutionary Ecology, Institute of Evolution and Ecology, University of Tuebingen, Tuebingen, Germany; University of Western Ontario, Canada

## Abstract

Sexual selection, differences in reproductive success between individuals, continues beyond acquiring a mating partner and affects ejaculate size and composition (sperm competition). Sperm and seminal fluid have very different roles in sperm competition but both components encompass production costs for the male. Theoretical models predict that males should spend ejaculate components prudently and differently for sperm and seminal fluid but empirical evidence for independent variation of sperm number and seminal fluid volume is scarce. It is also largely unknown how sperm and seminal fluid variation affect future mating rate. In bedbugs we developed a protocol to examine the role of seminal fluids in ejaculate allocation and its effect on future male mating rate. Using age-related changes in sperm and seminal fluid volume we estimated the lowest capacity at which mating activity started. We then showed that sexually active males allocate 12% of their sperm and 19% of their seminal fluid volume per mating and predicted that males would be depleted of seminal fluid but not of sperm. We tested (and confirmed) this prediction empirically. Finally, the slightly faster replenishment of seminal fluid compared to sperm did not outweigh the faster decrease during mating. Our results suggest that male mating rate can be constrained by the availability of seminal fluids. Our protocol might be applicable to a range of other organisms. We discuss the idea that economic considerations in sexual conflict research might benefit from distinguishing between costs and benefits that are ejaculate dose-dependent and those that are frequency-dependent on the mating rate *per se*.

## Introduction

Sexual conflict is a potent driver of biological diversity [1]–[3] and can arise and be maintained when male and female fitness maxima for a given trait, or process, are not identical [1], [2]. For example, in insects, high mating rate often increases fitness in males but decreases fitness in females [3]. However, substantial ejaculate production costs [4]–[7] can lead to differential ejaculate allocation across successive matings because of ejaculate economics [4] or because of constraints on production [8]–[11]. There are several ways in which males allocate ejaculates. Males can allocate a fixed proportion of the currently available ejaculate to the female which, in the absence of ejaculate replenishment between matings, leads to ever smaller ejaculates [8]–[11] but does not necessarily lead to a reduction in male mating rate. Alternatively, a male may not adjust ejaculate volume and partitions the sperm and seminal fluid reserves into equal portions. Without ejaculate replenishment, this strategy results in a linear decrease in ejaculate reserves over successive matings [12], leading to ejaculate depletion, the inability to mate and a reduction in future mating rate. In the presence of competition for females, males may tailor their ejaculate in strategic ways [13].

Most studies examining ejaculate allocation, ejaculate economics and their effect on future mating rate focus on one component of the ejaculate, sperm number [8]–[13]. Whether and how the availability of the other important component, the seminal fluid, shapes male mating decisions has received little attention. Recent ejaculate allocation theory predicts that sperm and seminal fluid allocation can evolve independently and influence each other in future matings [14], [15]. One model assumes future male mating rate is not restricted by this allocation, other than by reduced male survival after high ejaculate investment [14]. The other model makes the strict assumption that investment in sperm is inversely related to investment into seminal fluid but does not consider the effects on mating rate [15]. Experimental evidence on independent allocation of sperm and seminal fluid is scarce, as is its effect on mating rate. Humans can show substantial variation in the sperm to seminal fluid ratio per ejaculate. However, detailed investigations are restricted to two taxa, three fly and one snail species and show conflicting results in important details. In *Drosophila melanogaster*, five matings in rapid succession lead to a depletion of seminal fluids but not of sperm [16], [17]. In this and another *Drosophila* species the male accessory glands (the production site of seminal fluids) but not the testes, evolved to larger size under increased mating rate (or sperm competition) [16]–[19] and the production of seminal fluid, not sperm was upregulated before an anticipated high mating rate [20]. These studies suggest an evolutionary advantage to males providing more seminal fluids but not more sperm and thereby suggest independent allocation of sperm and seminal fluid. Whether seminal fluid depletion in *D. melanogaster* affects future mating rate is unclear: While one study mentioned that males continued to mate when they were depleted of seminal fluids [17], another study demonstrated that nutrition affected seminal fluid function in the form of ejaculate defence but did not affect mating propensity [21]. Both studies suggest seminal fluid availability plays no role for future mating rate. By contrast, Bangham et al. [22] reported that male *D. melanogaster* that mated at higher rates showed faster recovery of accessory gland size than males with lower mating rate. While it is not clear whether males that mated more often had larger glands originally or showed stronger gland replenishment, this study suggests a correlation between seminal fluid availability and mating rate. In the stalk-eyed fly, *Cyrtodiopsis dalmanni*, Baker et al. [23] reported that the replenished size of the accessory glands, but not the testes, were positively correlated to previous mating rates and Rogers et al. [24] later showed that repeated mating only affected accessory gland size, not testis size. In hermaphroditic pond snails *Lymnaea stagnalis*, copulation partially depleted the accessory gland but not the sperm reservoirs and male mating behaviour is generally triggered by the capacity of the accessory (prostate) gland but not the sperm reservoirs [25]. By contrast, sperm numbers were modulated depending on the type of recipient but the amount of seminal fluid was not [26].

In summary, there is some empirical evidence that sperm and seminal fluid can be varied independently but it is not clear whether such variation might feed back to mating rate. As these relationships are central to models of ejaculate allocation, sperm competition and sexual conflict we tested the predictions that a) available sperm and seminal fluid volume can be allocated independently of one another and b) that the limiting component restricts mating rate.

## Methods

### Study animals

**Figure 1:**
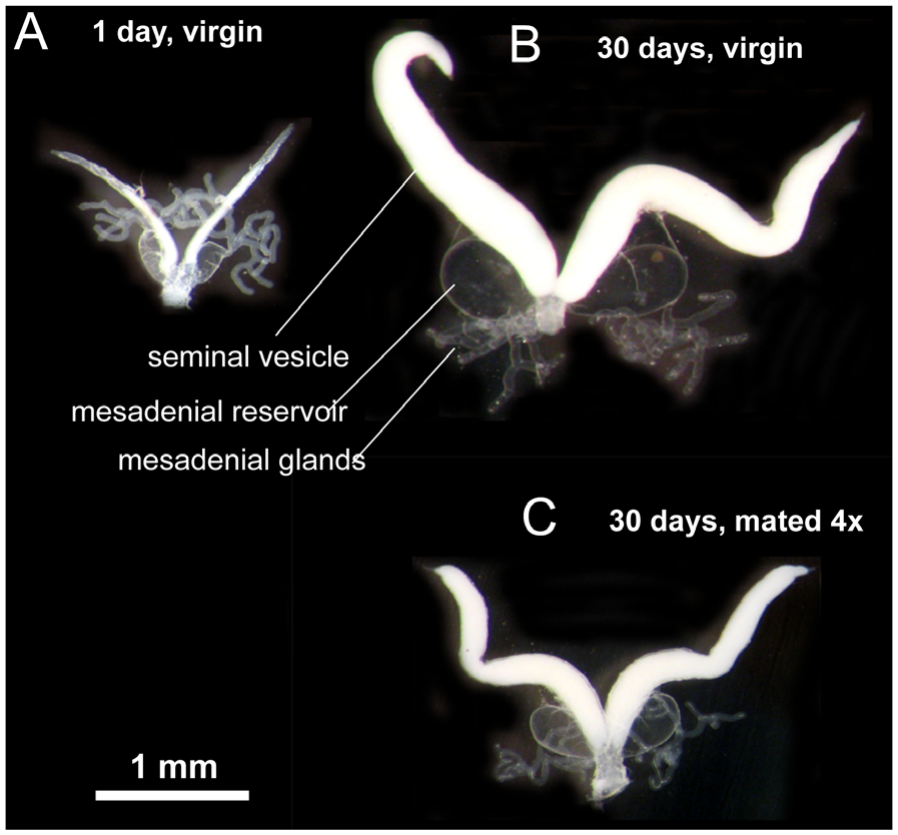
Anatomy of the reproductive tract of the common bedbug *Cimex lectularius*. Photograph showing the reproductive organ of a male (*a*) 3 hrs after eclosion, (*b*) 1 month after eclosion and (*c*) after four successive 60 s - matings one month after eclosion and sexual isolation. Fig. 1b from ref. [27].

Common bedbugs *Cimex lectularius* are well suited to study independent allocation of sperm and seminal fluid and their effect on future mating rate: males have discrete separated and easily measurable storage structures for sperm and seminal fluid ([27], Figure 1). Males have previously been demonstrated to allocate total ejaculate volume economically [28]. It has also been shown that males control the mating rate [29], [30] allowing experimental matings to be carried out without female behaviour limiting the time between ejaculates and therefore reducing the influence of ejaculate replenishment. In bedbugs, mating *per se* causes female costs [31]–[33] not ejaculate components (which are beneficial: [34]).

Individuals of an established laboratory population were cultured, and virgin individuals generated, as previously described [29], [33]. All individuals were fed every seven days unless otherwise stated.

### General protocol

We designed a stepwise protocol, assuming that any currently available ejaculate volume to a male, V, consists of a minimum ejaculate volume below which mating is impossible, V_0_, additional ejaculate volume is portioned (V_m_), across the number of future matings m:
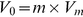
(1)


We measured the age-related increase in mating rate and ejaculate volume and determined the smallest sperm volume V_S_ and seminal fluid volume V_F_ at which males mated (V_S0_ and V_F0_). We then used V_m_ for sperm (V_Sm_) and seminal fluid (V_Fm_) to predict whether m_max_ results in V_0_ for sperm (V_S0_) or for seminal fluid (V_F0_) and empirically tested it. In the last step we tested whether mating-exhausted individuals replenished V_S_ and V_F_ at similar rates.

### Measuring mating rate

All matings were staged in a plastic petri dish (3 cm diameter) lined with filter paper and the lid closed. Each male was successively presented with one freshly fed female that was either virgin or had not mated for one day. Both types of females are void of sperm in the female copulatory organ, the spermalege [35] and it has been demonstrated that males transfer sperm at full rate to such females, rather than at the reduced rate seen during copulations with recently mated females [28]. In order to reduce variation in sperm volume because of copulation duration and to increase comparability to other studies (e.g. [30], [33], [34], all copulations were interrupted after 60 seconds (so-called ‘standard matings’ (SM)). Overall ejaculate transfer was linearly related to copulation duration [28].

We measured mating rate as the number of SMs a focal male performed in one hour. We choose one hour because in several hundred previous observations mating activity declines substantially thereafter (K. Reinhardt and R. Naylor, unpubl. observations). We assumed that there is little replenishment of sperm or seminal fluid over this period (see also Results).

### Measuring ejaculate volume

The male genital tract consists of a pair of testes from which pairs of widened *vasa deferentia*, so called sperm vesicles, descend towards the ejaculatory pump. Close to each of the sperm vesicles is a reservoir filled with the clear seminal fluid from the accessory glands (mesadenial gland *sensu* [35]), which also lead into the ejaculatory pump (see Figure 1). We measured the size of the sperm vesicle and the seminal fluid reservoirs (see Figure 1) and estimated sperm density (in pilot studies we found increasing sperm accumulation leads to more dense sperm packing). The reproductive tract (without testes) was dissected out and placed in phosphate-buffered saline (PBS) under a cover slip bridge preparation. A picture of the entire tract was obtained at defined magnification and light regime (see below) using the software package OPTIMAS 6.0 attached to a digital camera (Pulnix). The area of the sperm vesicles as well as the accessory gland reservoir was traced from these images using OPTIMAS 6.0 and calculated using size-calibrated scale bars. The cover slip bridge was constant (2 cover slips ∼0.3 mm) such that area was linearly proportional to volume.

We estimated sperm density by using the average grey value of the traced sperm vesicle area. This grey value is the weighted average luminance value (WAL) of a specified area. It varies between 0 (black) and 255 (white). All measurements were carried out in a light-proof box with a constant intensity and position of artificial light. The latter was calibrated to give the same grey value prior to taking measurements (Note, it was not the black background in Figure 1). The mean WAL of a traced area (the mean grey value) was taken as a surrogate for sperm density. The composite measurement sperm density×sperm vesicle size was regarded as V_S_.

Two measurements each of 40 samples showed the size of the sperm vesicle, the seminal reservoirs and the grey value very repeatable: Analyses of variance (two-way mixed effect models) indicated that between 0.5% and 2.8% of the measurement variance was within a single sample (all non-significant). The intraclass correlation coefficients (repeatabilities) ranged from 0.79<r<0.94, all P<0.001.

All observations were carried out using SMs. This can pose a problem if one ejaculate component were delivered at a lower rate than the other at the beginning of the copulation, its volume would be artificially truncated by our 60 s restriction. We examined this issue by comparing the slopes of the relationship between V_S_ and V_F_. Using a generalized linear model (glm) procedure in S-PLUS we asked whether the type of mating (SM or *ad libitum* taken from the populations described below) explained a significant part of the relationship between V_S_ and V_F_. Square-root transformation of V_S_ prevented overdispersion of the data.

### Measuring V_0_: Reproductive parameters in relation to male age

Freshly eclosed (±0.5 days), individually reared males were assigned to age cohorts. From these males, we identified i) age-related changes in V_S_ and V_F_ and ii) the age of first mating. The V_S_ and V_F_ at which males start to mate provides measures of V_S0_ and V_F0_. Mating rate, V_S_ and V_F_ were measured as described above. Because sampling of V_S_ and V_F_ is destructive we used a different sample of males kept under identical conditions in the environmental chambers. Recently eclosed individuals (0 to 3 hours) were used to generate data for adult age of 0 days.

### Measuring V_M_: Decline of ejaculate volume over mating rate

We measured the depletion of ejaculate components by cross-sectional comparisons of the volume of the sperm vesicle, the seminal reservoirs and the sperm density after successive matings. We used 20-day old virgin males and randomly allocated them to 4 treatment levels of 1, 2, 3 or 4 matings. These trials were carried out as individual male-female pairings in the mating arenas described above.

### Verifying the decline in ejaculate volume in small populations

Observations of dispersing bedbugs suggest that infestations are likely to be founded by single, or few, individuals [36] resulting in inbred populations. Given that mating behaviour and sperm allocation can change dramatically under such conditions (e.g. [37]–[39]) we tested whether ejaculate depletion can be observed under conditions that more closely resembled inbred infestations. We used sixteen iso-female lines that had been fed weekly but otherwise were left undisturbed to reproduce continuously for six months in 60 ml plastic containers, resulting in high densities (10 to 32 adults) as observed in heavily infested houses [40] and sex ratios between 41 to 92% females, also similar to natural conditions [40]. Earlier research has shown that fed females cannot avoid male copulatory attempts [30] and that the mating rate in a population will increase after feeding [29], [36]. We therefore fed all sixteen populations and compared V_S_ and V_F_ of a randomly drawn male before feeding with that of a randomly drawn male 24 hours after feeding. The period of 24 h should result in almost maximum mating rate [29]. Note that this trial was carried out to test whether notable declines in V_S_ and V_F_ can be observed. These trials cannot be used to calculate V_m_ in the ‘field’ because we were unable to restrict mating durations to that of SMs.

### Does m_max_ * V_m_ result in V_S0_ or V_F0_?

Twenty-nine males were isolated from a large stock population and left for 24 h. Individual males were then successively provided with freshly fed virgin females in a mating arena and allowed one SM with each. The number of SMs was recorded until the male made no mating attempt for 60 minutes. These males were either allocated to study ejaculate replenishment (see below), or immediately dissected in order to measure V_S_ and V_F_.

### Ejaculate replenishment

Males that had refused to mate (see above) were left in isolation for 1, 2, 3, 4, 7 and 10 days. V_S_ and V_F_ were then measured.

## Results

### V_0_: Reproductive parameters in relation to male age

**Figure 2:**
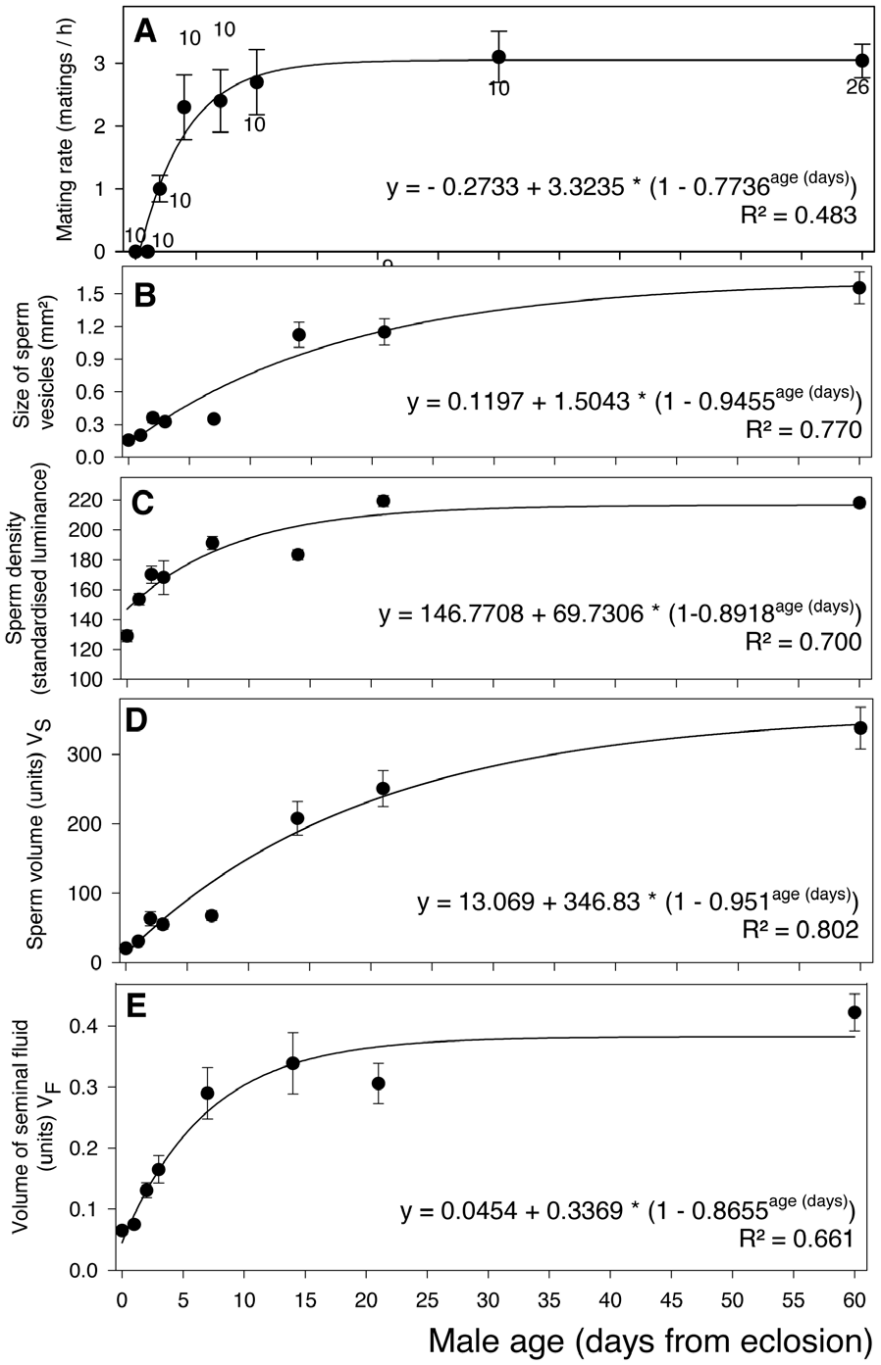
Age-related reproductive parameters of the common bedbug *Cimex lectularius*. Mating rate (a), size of sperm vesicles (b), sperm density (c), and the volume of sperm (V_S_) (d) and seminal fluid (V_F_) (e) increases with male age in the common bedbug. V_S_ (d) is a composite measure of sperm vesicle size and sperm density. Circles represent means, error bars = 1 standard error. Sample sizes are given near the mean values for each age group. Samples sizes in c–e are identical. Equations in graphs represent regression parameters from curve fitting procedures.

Mating rate increased from day 0 to day 3 levelling off around day 15 at a mean of 3.1 SMs per hour (range 1 to 6) (Figure 2a, F = 43.40, df = 2,94 p<0.0001). Similar increases was observed in V_S_ (Figure 2d, F = 50.12, df = 2,65 p<0.0001) and V_F_ (Figure 2e, F = 74.72, df = 2,65 p<0.0001), as well as in sperm vesicle size (Figure 2b, F = 107.42, df = 2,65 p<0.0001), and sperm density (Figure 2c, F = 61.59, df = 2,65 p<0.0001). Mean male mating rate showed the highest variability of the five parameters (Figure 2a). Mean male mating rate was positively correlated to the V_S_ (r = 0.775, p = 0.024, n = 8) [as well as its components, sperm vesicle size (r = 0.77) and sperm density (r = 0.86)], but had the closest correlation with V_F_ (r = 0.91, p = 0.002). The smallest average volume at which males started to mate was at day 2, when V_S_ = 63 units and V_F_ = 0.131 mm^2^, respectively.

### V_m_: Decline of ejaculate volume over mating rate

**Figure 3:**
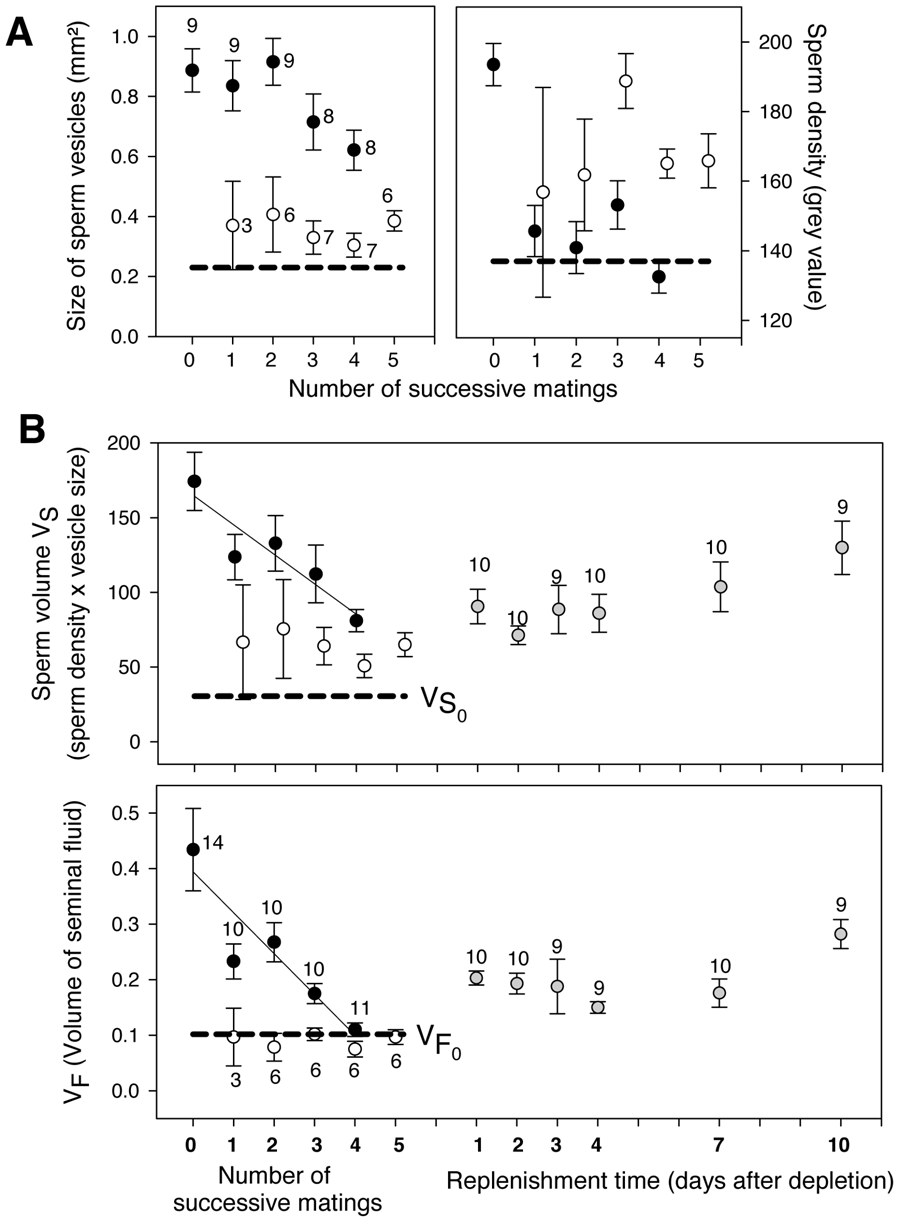
Ejaculate allocation over successive matings in the common bedbug, *Cimex lectularius*. The data points show cross sections of males after a number of matings in a) the size of sperm vesicles and sperm density, b) sperm volume (V_S_) and c) seminal fluid volume (V_F_). Black circles show sexually isolated males after a number of prescribed matings. The solid lines show linear least-square curve fittings. White circles show males drawn at random from a population that were examined after they stopped mating. Stippled lines show the value of the respective parameter at eclosion, i.e. a value below which mating is impossible. Grey circles show the replenishment of V_S_ and V_F_ of ejaculate depleted males in relation to time since sexual isolation. Circles represent means, error bars 1 standard error. Sample sizes are stated near the mean values. Sample size in Figure 3a and b are identical.

Sperm vesicle size and sperm density decreased in a different way over successive matings (Figure 3a): Sperm density showed the largest decrease at the first mating, while sperm volume showed the largest decrease from the third mating. Their composite measure, V_S_, and V_F_ showed a more even decline, amounting to a male mean of V_Sm_ = 19.78 units and V_Fm_ = 0.0736 units (Figure 3b,c), or 12.03% of the available V_S_ and 18.7% of the available V_F_. Assuming a decline at these rates, V_S0_ would be reached after 7.25 matings, V_F0_ after 4.48 matings. Therefore, during SMs V_F_ decreases more rapidly than V_S_.

**Figure 4:**
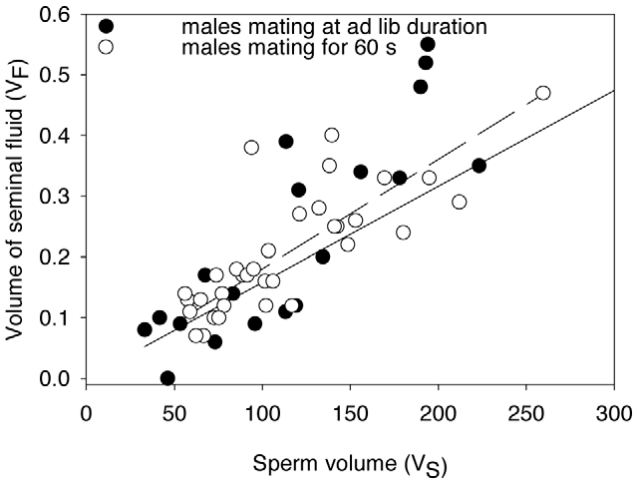
Relationship between interrupted and non-interrupted matings in the common bedbug *Cimex lectularius*. Correlation between the volume of sperm V_S_ and seminal fluid V_F_ stored by males after mating *ad libitum* and after mating for 60 s (the standard duration used in this paper, SM). The fact that SM did not have a smaller slope than *ad libitum* matings shows that SM did not result in truncation of seminal fluids.

Mating type did not explain a significant part of the relationship between V_S_ and V_F_ (Figure 4) (slope mating type: 0.0253±0.021 (s.e.), t = 1.232, df = 50 p = 0.888). We, therefore, conclude that SMs and *ad libitum* matings do not differ in the rate sperm and seminal fluid are delivered.

### Ejaculate decline in small populations

On average, V_S_ decreased by 75.52±25.15 (s.e.) (−29.1±12.2%) (t_paired_ = 3.003, df = 1,15, p = 0.009), V_F_ by 0.085 mm^2^±0.038 (s.e.) (t_paired_ = 2.24, df = 1,12, P = 0.045) (−21.8±14.2%). None of the males with access to a variable number of freshly fed females were sperm or seminal fluid depleted. Their mean V_S_ (85.67±9.8 (s.e.)) and V_F,_ (0.17±0.02 (s.e.)) would allow for a mean of 3.23 or 1.4 future matings, respectively. Neither V_S_ (r: −0.025) nor V_F_ (r = −0.16) were correlated to sex ratio (both p>0.5). We conclude that measurable declines in V_S_ and V_F_ take place in small inbred populations. These declines exceed 1 SM but despite sufficient time for further matings, males did not deplete their V_S_ and V_F_.

### Does m_max_ * V_m_ result in V_S0_ or V_F0_?

Randomly drawn stock males mated one to five times (overall mean of 3.18±s.d. 1.28) when exposed to females. The variation between males that stopped mating was particularly small, and independent of the number or previous matings (empty circles in Figure 3), in V_F_ (Figure 3c) and to a lesser degree for sperm vesicle size (Figure 3a), V_S_ (Figure 3b) and sperm density (Figure 3a). The observed mean volumes (V_S_ = 63, V_F_ = 0.089) would allow for a further 2.24 and 0.32 matings respectively. Examining those males in more detail revealed that in three males V_F_ at refusal was even lower than V_0_ observed at eclosion, but no male had a V_S_ lower than at eclosion. When we, therefore, relaxed the criterion for V_0_ and considered V_0_* the mean between the smallest volume that allowed a male one future mating (i.e. V_m(max−1)_) and the total minimum at eclosion (V_S_ = 30.5, V_F_ = 0.1017), the V_S_ of four of 29 males (14%) but V_F_ of 17 of 27 males (63%) was below V_0_* (Fisher's exact test, p = 0.0002). In addition, all individuals whose V_S_ = V_S0_* also showed V_F_ = V_F0_* suggesting that in no case did V_S_ depletion alone cause mating refusal.

### Ejaculate replenishment

The reproductive organs of non-maters replenished relatively slowly (Figure 3b,c). After ten days in sexual isolation none of the ejaculate parameters, other than sperm density, had reached the level observed prior to mating (Figure 3b,c). For example, after 10 days V_S_ had reached 129.93 units (Figure 3b), allowing for an average of 5.51 possible future matings. At the same time, V_F_ had reached 0.282 units (Figure 3c), corresponding to 2.95 possible future matings. This comparison suggests that V_F_ replenishment, although slightly faster than V_S_ replenishment, does not outweigh the more rapid decrease during successive matings.

## Discussion

The seminal fluid reserves of bedbug males declined faster than sperm reserves and we empirically confirmed that males stopped mating when they were depleted of seminal fluid but not sperm. The faster decline in seminal fluid reserves compared to sperm was not outweighed by faster replenishment of sperm reserves. Taken together, these results suggest that bedbug male mating rate is limited by the availability of seminal fluid. Our results have consequences for studies of postcopulatory sexual selection, sexual conflict, inbreeding and research into age-dependent reproductive decisions.

### Postcopulatory sexual selection

Seminal fluids have become a focus of research into postcopulatory sexual selection because of the diversity of their components [27], [41], [42] and because of the postcopulatory benefits they provide to the male, female or both [15], [42]–[44]. Although male postcopulatory benefits have been well described there seem to have been few attempts to link them to precopulatory processes such as the ability to re-mate. Here we have shown that the low availability of seminal fluids, not sperm, constrains male mating rate. We found that low seminal fluid availability resulted in low mating rate in a number of contexts. First, over male age, mating rate was closer correlated to the volume of seminal fluid reserves than sperm reserves. Second, sexually active males performing standard matings were depleted in seminal fluids before they were depleted in sperm. Third, in inbred family groups with the opportunity to mate for *ad libitum* durations, it was male seminal fluid reserves that constrained future mating rate. It is possible that in these contexts mating rate restriction is important in other organisms, too. The protocol presented here might be a useful tool to examine this possibility.

Recent theoretical models [14], [15] suggest that differential allocation of sperm and seminal fluid is particularly advantageous with respect to mating order. If the previous seminal fluid, but not sperm, expenditure of a male restricts his future re-mating ability, one may expect stronger selection on internal signals of accessory gland than sperm reservoir capacity. Indeed, in a snail the size of the accessory gland (prostate gland), not the size of the sperm reservoirs triggers male mating rate via a neuronal response [25]. The generality of these models remain to be tested. For example, in the presence of rival sperm bedbug males reduce, not increase, the transfer of ejaculate volume [28]. While the latter study suggests that the sperm component may be decreased, rather than increased, future studies should examine the change in sperm to seminal fluid ratio with respect to mating order. Our protocol may also be useful for that task.

### Estimating male harm in sexual conflict

The relatively well-known aspects of conflict and cooperation between the sexes in the bedbug means this species is a good model for this aspect of sexual selection [27]–[36], [44]–[46]. In the bedbug, the male-induced mating rate is currently calculated as approximately 20-fold exaggerated because one mating every four weeks allow maximal fecundity but males mate five times per week [29]. Our results show that costs based on one-week long observations may not necessarily be projected to the female's entire lifespan: after 5 matings, ejaculate supplies were not replenished within a week and after ten days had only recovered to a point of allowing approximately 1.5 matings. Consequently, if males mate 5 times per week+1.5 times during the following 10 days (i.e. 6.5 matings/17 days = 0.38 matings per day), this is only slightly more than half the current estimate of 5 matings per 7 days (0.71 per day). We, therefore suggest that the current estimate of a 20-fold male mating exaggeration over the female optimum should be reduced to an average of 11-fold. This estimate should be followed up by studies refining the period that once-mated females can lay eggs for, which may exceed four weeks (summarized in [36]).

When considering the effect of mating rate on female fitness, it might be useful to consider whether female costs of mating are caused by the ejaculate components in a dose-dependent manner. Mating rate *per se* will be important when female costs increase with the number of matings, such as copulatory damage [47]–[49] or infection with STDs [50]–[53]. By contrast, some costs may be dose-dependent such as the cumulative toxicity of male accessory gland fluid [43] or costs of sperm storage [53], [54] that depend on total ejaculate volume transferred, or copulation duration, not on the number of matings *per se*. In the bedbug, ejaculate volume has a positive effect on females [34] but mating has a negative one [32], [33]. Our study adds to that picture by showing that ejaculate substances with a beneficial effect on females are in short supply in the male but that this short supply also reduces the projected male mating rate.

### Mating rate under inbreeding

We have shown that male mating activity can also be demonstrated in small semi-natural populations by a decrease in V_S_ and V_F_ after mating opportunity. However, although males were neither time constrained nor limited in mating partners, males drawn at random from our populations were not sperm or seminal fluid depleted. This was in contrast to the situation when a considerable number of males were seminal fluid depleted when individually exposed to females. Presently, we have no robust explanation for this difference. It is possible that males avoid mating with familiar [30] or related females. In the latter case the evolution of reduced harm, but not of reduced female counter-adaptations to male harm is predicted [55]. This may happen by males either reducing their overall mating rate or, because the net ejaculate effect is positive for females [34], by transferring more ejaculate per mating. The former possibility seems more likely for two reasons. i) The harm to the female is dependent on the frequency of mating, ii) the reduction in V_S_ and V_F_ after male mating opportunity in the small populations was smaller than expected, not larger.

### Male age, ejaculate accumulation and sperm age

Unlike some insect species where males eclose with a fixed ejaculate volume that is tailored across several matings (Lepidoptera: [56]; Hymenoptera: [57]), we show here that bedbug males do not eclose with sperm reserves. From eclosion onwards, sperm and seminal fluid volumes accumulate in virgin individuals in a logarithmic way indicating their production rate increases linearly every day until ca. 20 days. Beyond 20 days, ejaculate volume increased slightly or stayed the same. It is not clear whether ejaculate volume ceases to increase because of feedback mechanism(s) related to the capacity of the organ or whether ejaculation production is constant beyond a certain age. The fact that ejaculate replenishment following repeated matings of 20 day old males showed a much slower increase in ejaculate production supports to the latter notion. The relatively sudden changes in ejaculate volume before day 1 and after day 7 of replenishment (see Figure 3) may be related to a release of seminal fluid reserves from the accessory (mesadenial) glands (cf. Figure 1) and a feeding event, respectively.

The ejaculate volume of males did not decrease beyond a certain age, until we finished the observations at day 60. This suggests that in the absence of mating opportunities, males do not resorb unused sperm. If they do not, this suggests that some sperm cohorts are as old as the male [58]. If males lack the ability to resorb sperm, they may be constrained by the detrimental effects of ageing in such accumulated sperm cells [58], [59]. At day 50–60 of sexual isolation, male bedbugs spontaneously start to inseminate other males if no females are present [45], [46]. Among other explanations, this behaviour has been proposed to serve as a male strategy to get rid of accumulated aged sperm [58]. Provided the accumulated seminal fluid does not deteriorate, our protocol could be used to test the idea that males only transfer sperm but not seminal fluid to other males.

### Future research

Given that seminal fluids are a mixture of a large number of substances [27], [41], [42], future studies may be directed to study changes of individual components of the seminal fluid within and across matings. For example, in *Drosophila melanogaster* the transferred volume of two specific seminal proteins declined differently across successive matings [60]. The depletion, and therefore possibly also the strategic allocation, of specific seminal fluid components may also be influenced by the variation between males. For example, more than 100 substances were found in the seminal fluid of the bedbug [27]. One of them, the immune active substance lysozyme, showed substantial concentration differences between males [44].

We propose that the function of individual components of the seminal fluid may also inform us about their relationship to future mating rate. If the male benefits of seminal fluid transfer accrue in a dose-dependent manner such as fecundity stimulation [15], [42], selection on future re-mating is likely to be weaker because dose-dependent benefits could be achieved by prolonged seminal fluid transfer to the same female. Seminal fluid benefits to males that accrue in relation to the frequency of mating, such as aspects of sperm activation [35], or antimicrobial [44] and antioxidant [27] protection of sperm may be more likely to be economised across matings.

Finally, one future area of research may concern a beneficial role of sperm accumulation in the male. In most animals, most sperm in an ejaculate do not fertilise the egg. However the non-fertilising sperm might support the function of fertilising sperm, such as demonstrated for sperm heteromorphic species [61] or via positive density-dependent sperm survival or reduced sperm ageing [58]. If non-fertilising sperm - the majority of sperm in an ejaculate - have a protective function, they would occupy one functional role of seminal fluid. This may affect theoretical predictions about sperm and seminal fluid allocation [14].

In conclusion we provide empirical evidence for the notion [14] that sperm and non-sperm allocation can evolve independently. Their respective effect on future mating rate should also be considered in models of ejaculate economics [4].
